# Mobile-assisted academic vocabulary learning with digital flashcards: Exploring the impacts on university students’ self-regulatory capacity

**DOI:** 10.3389/fpsyg.2023.1112429

**Published:** 2023-04-03

**Authors:** Tahereh Boroughani, Nastaran Behshad, Ismail Xodabande

**Affiliations:** ^1^College of Education and Human Development, Texas A&M University, College Station, TX, United States; ^2^Faculty of Percian & Foreign Language, University of Tabriz, Tabriz, East Azerbaijan, Iran; ^3^Department of Foreign Languages, Kharazmi University, Tehran, Iran

**Keywords:** mobile-assisted vocabulary learning, academic vocabulary, digital flashcards, self-regulation, EAP, academic literacy, Quizlet

## Abstract

With the global rise in international journals over the past decades, successful communication in science largely hinges upon developing competency in using English as the academic *lingua franca*. Accordingly, one aspect of developing academic literacy entails helping university students learn a group of medium-frequency and cross-disciplinary words (i.e., core academic vocabulary) employed extensively to describe abstract processes and organize rhetorical aspects of academic discourse. The current study aimed to investigate the contribution of mobile-assisted vocabulary learning with digital flashcards in scaffolding academic vocabulary learning and self-regulatory capacity development among university students. The participants were 54 Iranian university students selected based on their availability in the study context. The participants were assigned to an experimental group (*N* = 33) and a control learning condition (*N* = 21). Those in the experimental group used digital flashcards (i.e., Quizlet) to learn academic words in a recently developed core academic wordlist (i.e., NAWL), and the control group used traditional materials (wordlists) to learn the same vocabulary items. The participants’ vocabulary knowledge and self-regulatory capacity for vocabulary learning were tested before and after the treatments. The findings indicated that although both groups improved their vocabulary knowledge and self-regulatory capacity after 4 months, the experimental group outperformed the control group in both measures, and the effect sizes of the observed differences were very large. Consequently, the study provided empirical evidence for the effectiveness of mobile-assisted vocabulary learning over traditional materials in developing academic literacy. The findings also indicated that using digital flashcards for vocabulary learning improves university students’ capacity for undertaking self-regulated vocabulary learning. The implications of these findings for EAP programs are highlighted.

## Introduction

With the global dominance of English as the academic *lingua franca* (Hyland, 2009, 2022), university students and researchers increasingly need to improve their knowledge of this language to read published studies in their fields and also to publish their research in international journals (Li and Flowerdew, 2020). Accordingly, along with the proliferation of English for Academic Purposes (EAP) programs around the world (Hyland, 2006, 2013), developing university students’ academic literacy is attracting increased attention (Muresan and Orna-Montesinos, 2021; Li, 2022). One critical aspect of academic literacy is learning and using academic words (Coxhead, 2000). Defined as those words that are used with higher frequency in the discourse of academia (rather than non-academic discourse; Gardner and Davies, 2014), academic vocabulary is employed extensively for writing about abstract ideas and processes in science (Nagy and Townsend, 2012). These words are also used extensively for structuring and framing rhetorical organization of academic texts (Paquot, 2010; Coxhead, 2019). Recent corpus-based investigations into academic discourse showed that academic words constitute a considerable proportion of academic texts such as research articles and textbooks, with coverage ranging from 6 to 14% of all words depending on different academic word lists (Coxhead, 2000; Browne et al., 2013b; Gardner and Davies, 2014). Some widely used academic words in English include repertoire, obtain, distribution, parameter, aspect, dynamic, impact, domain, publish, and denote (Browne et al., 2013b).

Scaffolding the processes involved in learning academic vocabulary among English as a Foreign Language (EFL) students is essential for some reasons. First, previous research indicated that learning vocabulary and developing lexical competence in a foreign language is a long-term process that takes many years (Webb and Chang, 2012; Rahmani et al., 2022; Zakian et al., 2022). Accordingly, there is a need for pedagogical interventions to facilitate vocabulary knowledge development among EFL learners (Webb and Nation, 2017). Second, learning (and using) academic words are associated with a considerable learning burden for EFL university students (Evans and Morrison, 2010, 2011; Xodabande et al., 2022; Boroughani et al., 2023). In this regard, there is a growing need for a systematic focus on covering these words in EAP programs regardless of the learning context (Coxhead, 2018, 2019; Xodabande and Boroughani, 2023). Additionally, the expanding body of knowledge in corpus-based studies of research articles as the pre-eminent genre in academic discourse has shown the significant role and the importance of academic words in scientific communication (Martínez et al., 2009; Lei and Liu, 2016; Xodabande et al., 2022). It has also been argued that using academic words is closely related to professional identity construction in writing for publication (Hyland and Tse, 2007; Green and Lambert, 2018; Roesler, 2021). Consequently, research on identification and teaching academic vocabulary remained an active area of inquiry, being mainly concerned with addressing pedagogical challenges in EAP programs (Coxhead, 2019).

Over the past two decades, significant expansion in digital technologies provided us with new tools and resources to address EFL university students’ vocabulary learning needs (Lin and Lin, 2019; Burston and Giannakou, 2021; Hao et al., 2021; Yang et al., 2021; Yu and Trainin, 2022). One main line of research within this literature investigated the learning outcomes of using mobile devices for vocabulary learning. Accordingly, the findings from a large number of studies indicated that mobile-assisted learning is generally more effective compared to the learning conditions involving traditional materials (Mahdi, 2017; Lin and Lin, 2019). Moreover, accumulated empirical evidence in this area suggests that mobile-assisted vocabulary learning effectively develops receptive and productive knowledge of target words (Li and Hafner, 2022; Xodabande et al., 2022). Additionally, the related studies pointed to both short- and long-term learning impacts of mobile-assisted instruction (Xodabande and Atai, 2022). Considering the effectiveness of flashcards (or word cards) in fast and effective vocabulary learning (Nakata, 2019; Lei and Reynolds, 2022), mobile-assisted academic vocabulary learning *via* digital flashcards is gaining popularity (Dizon, 2016; Xodabande et al., 2022; Xodabande and Atai, 2022; Yüksel et al., 2022; Boroughani et al., 2023; Xodabande and Boroughani, 2023). Collectively, these studies showed that digital flashcards provide university students with affordances for augmenting their vocabulary knowledge more systematically and beyond the physical and temporal restraints of the classrooms. Nevertheless, although most studies were conducted in self-regulated and beyond the classroom learning environments, scant attention has been paid to the contribution of mobile-assisted vocabulary learning on university students’ self-regulatory capacity (Tseng et al., 2006; Tseng and Schmitt, 2008; Fathi et al., 2018; Lei et al., 2022; Teng et al., 2022). As self-regulatory capacity is a key factor in strategic vocabulary learning, research in this area has considerable potential to improve second language literacy (Boroughani et al., 2023).

In educational psychology, self-regulated learning (SRL) theory is primarily concerned with factors and processes such as metacognition, motivation, and strategic action that are significantly important in learning pre-specified content (Zimmerman, 2002; Brenner, 2022). Additionally, helping students to become self-regulated learners is now considered an essential goal for modern educational systems (Zimmerman, 2008). Relatedly, SRL is gaining increased recognition as a defining component in language learning (Zhang et al., 2019; Teng and Zhang, 2020). Research focusing on the relationship between vocabulary learning and SRL among EFL students indicated that their capacity to engage in SRL contributes significantly to developing their lexical competence (Choi et al., 2018; Fathi et al., 2018; Şahin Kızıl and Savran, 2018; Chen et al., 2019). For example, Chen et al. (2019) investigated the role of a vocabulary learning application with a mechanism for self-regulated learning that aimed to help learners to enhance their SRL abilities, learning performance, and motivation. The study revealed that those learners who used the SRL mechanism attained better learning outcomes and improved motivation. Moreover, focusing on technology-assisted learning environments, Şahin Kızıl and Savran (2018) developed an instrument for measuring the self-regulatory capacity of EFL students for vocabulary learning. The study indicated that self-regulated capacity for using technology might be a valid predictor of EFL students’ success in learning vocabulary *via* a wide range of information and communication technologies.

Moreover, some studies investigated the impacts of mobile-assisted learning on language learners’ self-regulatory capacity. In this regard, Kondo et al. (2012) explored the impacts of mobile-assisted learning on Japanese university students’ self-regulated learning. The findings indicated that using mobile devices for language learning contributed to increased motivation among learners and improved their self-regulatory capacity. In another study, Zheng et al. (2018) developed a mobile-based self-regulated system for supporting university students’ self-regulated learning skills. The study findings revealed that the developed system significantly improved the students’ skills for self-regulated learning without much impact on the cognitive load associated with learning tasks. Furthermore, Ferreira (2014) examined young English language learners’ self-regulated learning in a technology-supported environment. The findings of the study showed that those students who received technology-supported training had better performance in vocabulary learning tasks. Accordingly, the existing literature suggests that not only self-regulatory capacity is a good predictor of success in technology-assisted vocabulary learning, the use of digital devices for vocabulary learning also impacts language learners’ self-regulatory capacity.

Despite this recent interest in exploring various aspects of self-regulated vocabulary learning among EFL learners, a number of gaps in the literature foreground the need for further empirical studies. First, the application of SRL theory to language education is a relatively recent phenomenon (Kim et al., 2015; Teng, 2022). The limited evidence in this area makes it difficult to see how such learning mechanisms contribute to developments in different language skills including second language vocabulary learning (Şahin Kızıl and Savran, 2018). Second, although research on mobile-assisted vocabulary learning expanded considerably (Lin and Lin, 2019), utilizing the affordances provided by mobile platforms for scaffolding academic vocabulary learning as a key factor in academic literacy development remained less explored (Xodabande and Atai, 2022). Additionally, considering the importance of vocabulary learning strategies in long-term and effective vocabulary knowledge development for EFL students (Pavii Taka, 2008), the contribution of well-established strategies such as digital flashcards for enhancing the self-regulatory capacity of EFL learners remained less explored (Fathi et al., 2018; Nakata, 2019; Lei et al., 2022; Lei and Reynolds, 2022). The present study aimed to address the abovementioned gaps by answering the following research questions:

1. Compared to traditional wordlists, is mobile-assisted learning with digital flashcards more efficient in promoting university students’ academic vocabulary?
2. Compared to traditional wordlists, is using digital flashcards strategy more effective in impacting students’ self-regulatory capacity for vocabulary learning?

## Method

### Participants

The participants of the study were 54 Iranian adult language learners (22 males and 32 females) selected based on their availability in the study context. The mean age of the students was 22, and the results of the Cambridge placement (Cambridge English, 2022) test indicated that most of them were at pre-intermediate (i.e., B1) level of proficiency in English based on the Common European Framework of Reference for languages (Council of Europe, 2001). Following Li and Hafner (2022), the participants were assigned to an experimental learning condition (*N* = 33) and a control group (*N* = 21) based on their preferences for using digital flashcards on their smartphones or paper-based materials (i.e., wordlists) for academic vocabulary learning. This allowed the students to select the type of learning material that was more appropriate for their vocabulary learning. Informed consent was obtained from the participants, and they were ensured regarding the confidentiality of the collected data and personal information during the study.

### Materials and instruments

The current study used New Academic Word List (NAWL) (Browne et al., 2013b) as a source for academic vocabulary in English. Developed based on a large corpus including research articles, non-fiction, student essays, and academic discourse [288 million words from Cambridge English Corpus (CEC)],^1^ NAWL contains 960 words and provides around 6% coverage in most academic texts. Considering the 86% coverage of general words in the academic subsection of the CEC (Browne et al., 2013a; Browne, 2021), these words contribute significantly to understanding academic discourse among university students. In order to learn NAWL items, the participants in the experimental group used NAWL flashcards designed in 19 fifty-word blocks for Quizlet.^2^ Those in the control group were given 19 word lists containing the same vocabulary items in the Quizlet flashcards.

The study used a number of testing instruments. First, the participants in both groups were tested using the New Vocabulary Levels Test (NVLT; McLean and Kramer, 2015). This standard and validated vocabulary test for English language learners was used as a criterion measure to ensure the homogeneity of the sample before the treatments. The range of possible scores for this test was between zero and 120. Second, for testing the knowledge of academic words before and after the treatments, the study used the New Academic Word List Test (NAWLT; Stoeckel and Bennett, 2020), which is a validated measure for assessing the receptive knowledge of the NAWL. The NAWLT has 40 items which is also the range of scores. Third, the study employed Self-regulating Capacity in Vocabulary Learning Scale (Tseng et al., 2006; Ziegler, 2015) that contains 20 items based on a six-point Likert scale (1 = strongly agree to 6 = strongly disagree). The responses were scored (based on the Likert scale) as a composite measure by adding the values for each item. Accordingly, the scores for this scale were between 20 and 120. The instrument is designed and validated for measuring five domains of language learners’ self-regulatory capacity in learning vocabulary: commitment control, metacognitive control, satiation control, emotion control, and environment control.

### Procedures and data analysis

Before implementing the treatments, the participants’ general vocabulary knowledge was tested to ensure the homogeneity of the two groups. Next, the study assessed the participants’ academic vocabulary knowledge and self-regulatory capacity for vocabulary learning. Following these initial assessments, the participants employed different materials for learning academic words. The treatment was implemented as an out-of-the-classroom learning activity during an academic semester. In this regard, the participants were asked to study the target words for a minimum of 2 h every week (or 20–25 min per day), and their progress in vocabulary learning was checked weekly. More specifically, the participants were asked to keep learning logs and record their vocabulary learning activities, including the number of studied words and the amount of time spent. The participants’ academic vocabulary and self-regulatory capacity were tested again after 4 months at the end of the semester. The obtained data in pre- and post-tests was analyzed using IBM SPSS software (version 25). In this regard, data analysis included obtaining descriptive statistics for the scores obtained by the participants in different learning conditions and conducting a multivariate analysis of variance (MANOVA; Pallant, 2016) to explore data for between-group differences.

## Results

As mentioned in the previous section, before implementing the treatments, the participants in both groups were tested using the NVLT (McLean and Kramer, 2015) and Self-regulating Capacity in Vocabulary Learning Scale (Tseng et al., 2006; Ziegler, 2015). The results of the *t*-test comparing the two groups based on mean scores pointed to no significant difference in their pre-existing vocabulary knowledge, t (52) = 0.15, *p* ≤ 0.886 (experimental = 66.64, *SD* = 9.84; control = 66.19, *SD* = 11.81). Accordingly, the sample was homogenous regarding their vocabulary knowledge. The results of the descriptive statistics for the scores obtained on self-regulatory capacity (SRC) and academic vocabulary knowledge test (VKT) are summarized in Table 1. As shown below, the observed differences in the scores were not much noticeable in the pre-tests. However, the results obtained on the post-tests pointed to considerable differences in the participants’ performances in the experimental and control learning conditions.

**Table 1:** Descriptive statistics.

		Group	*N*	Mean	Std. Deviation	Std. Error mean
Pre-tests	SRC	Experimental	33	63.36	10.431	1.816
		Control	21	60.71	6.791	1.482
	VKT	Experimental	33	13.55	2.762	0.481
		Control	21	12.76	3.300	0.720
Post-tests	SRC	Experimental	33	72.55	7.938	1.382
		Control	21	61.48	9.760	2.130
	VKT	Experimental	33	23.82	3.877	0.675
		Control	21	17.76	3.576	0.780

SRC: self-regulatory capacity; VKT: vocabulary knowledge test.

As data collected for the study included the experimental and control groups’ scores on academic vocabulary test and their self-regulatory capacities at two times (i.e., pre-test and post-test), the results were analyzed for within-subjects and between-groups differences. Table 2 shows the results obtained for the tests of within-subjects effects. Accordingly, data analysis indicated that developments in the participants’ self-regulatory capacity (*F* (1, 52) = 6.98, *p* ≤ 0.011, *η_p_^2^* = 0.118) and academic vocabulary knowledge (*F* (1, 52) = 140.92, *p* ≤ 0.001, *η_p_^2^* = 0.73) were significant from pre-test to post-test. These results indicated that all participants improved their self-regulatory capacity and academic vocabulary regardless of the treatments (i.e., digital flashcards and wordlists) they received. However, the analysis also pointed to significant interaction effects between time and group variables. These significant interaction effects mean that the developments in the experimental and control groups’ self-regulatory capacity and academic vocabulary knowledge were different.

**Table 2:** Tests of within-subjects contrasts.

Source	Measure	Treatment	Type III sum of squares	*df*	Mean square	*F*	Sig.	Partial Eta squared
Time	SRC	Linear	634.465	1	634.465	6.986	0.011	0.118
	VKT	Linear	1496.727	1	1496.727	140.92	0.000	0.730
Time*Group	SRC	Linear	454.909	1	454.909	5.009	0.030	0.088
	VKT	Linear	178.394	1	178.394	16.797	0.000	0.244
Error (Time)	SRC	Linear	4722.359	52	90.815			
	VKT	Linear	552.273	52	10.621			

Considering significant interaction effects reported in Table 2, the data were analyzed for between-groups differences (Table 3). In this regard, the findings revealed that before the treatments, there were no significant differences in the participants’ self-regulatory capacity for vocabulary learning (*F* (1, 52) = 1.064, *p* = 0.307, *η_p_^2^* = 0.020); and their knowledge of academic vocabulary (*F* (1, 52) = 0.887, *p* = 0.351, *η_p_^2^* = 0.017). However, the observed differences between the groups were significant after the treatments. Accordingly, the two groups performed differently with respect to their self-regulatory capacity for vocabulary learning (*F* (1, 52) = 20.85, *p* ≤ 0.001, *η_p_^2^* = 0.286); and knowledge of academic words in English (*F* (1, 52) = 33.224, *p* ≤ 0.001, *η_p_^2^* = 0.390). More specifically, the participants in the experimental group outperformed those in the control group in learning academic words and developing their self-regulatory capacity for vocabulary learning. The effect sizes of the differences were large based on criteria proposed by Cohen (1988).

**Table 3:** Tests of between-subjects effects.

Source	Dependent variable	Type III sum of squares	*df*	Mean difference (I–J)	Mean square	*F*	Sig.^b^	Partial Eta squared
Group	SRC (pre-test)	90.078	1	2.649	90.078	1.064	0.307	0.020
	VKT (pre-test)	7.879	1	0.784	7.879	0.887	0.351	0.017
	SRC (post-test)	1572.450	1	11.069^*^	1572.450	20.851	0.000	0.286
	VKT (post-test)	470.707	1	6.056^*^	470.707	33.224	0.000	0.390

Based on estimated marginal means. SRC: self-regulatory capacity; VKT: vocabulary knowledge test.

* The mean difference is significant at the 0.05 level.

b Adjustment for multiple comparisons: Bonferroni.

**Figure 1:**
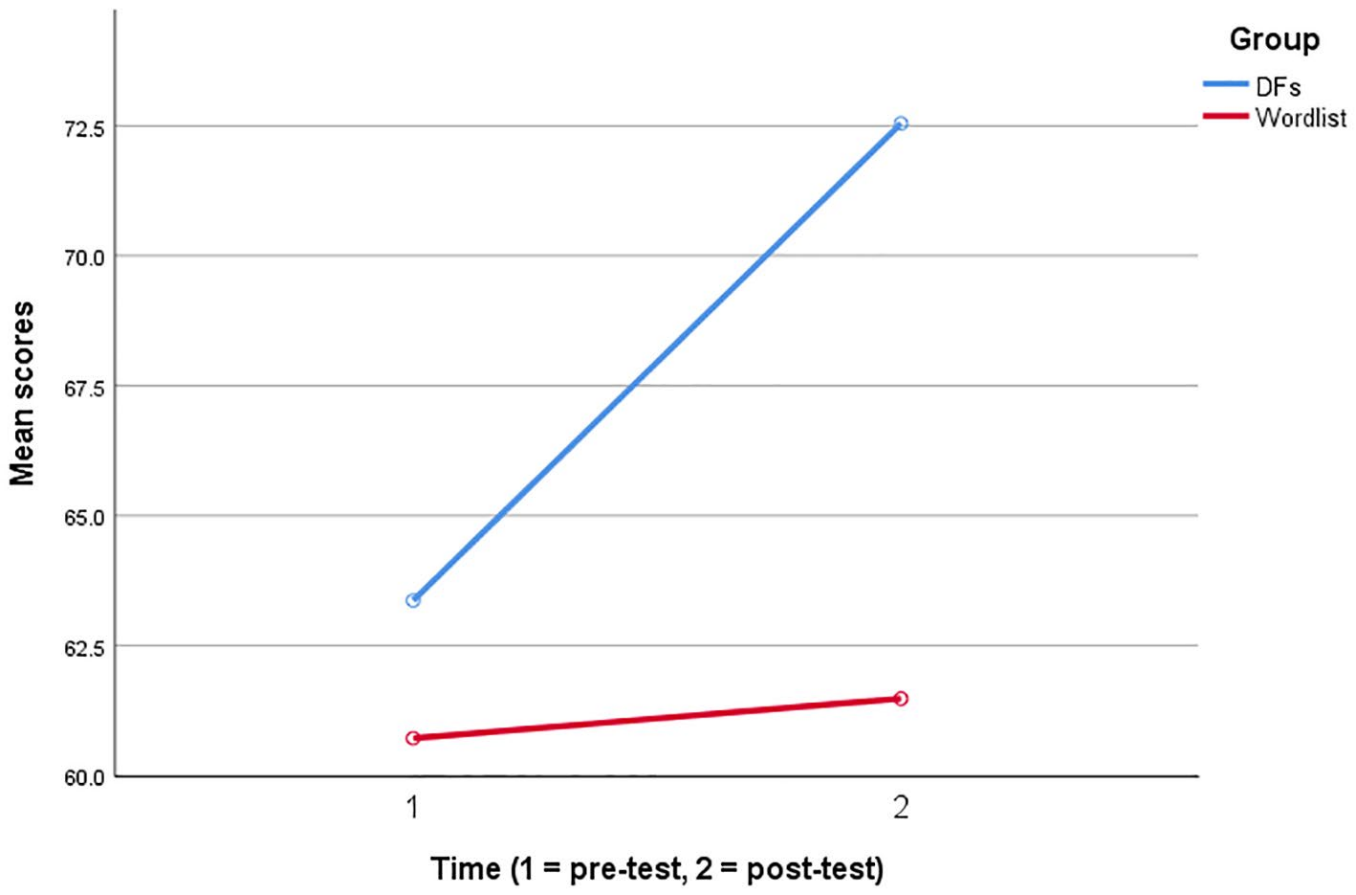
Estimated marginal means for self-regulatory capacity.

Figure 1 provides a visual representation of the estimated marginal means for self-regulatory capacity scores. As shown below, although the mean scores for the experimental (DFs) and control (wordlist) groups were close to each other before the treatments, the differences were significant in the post-test. Additionally, both groups improved their capacity for self-regulated vocabulary learning; however, using DFs resulted in more improvements.

**Figure 2:**
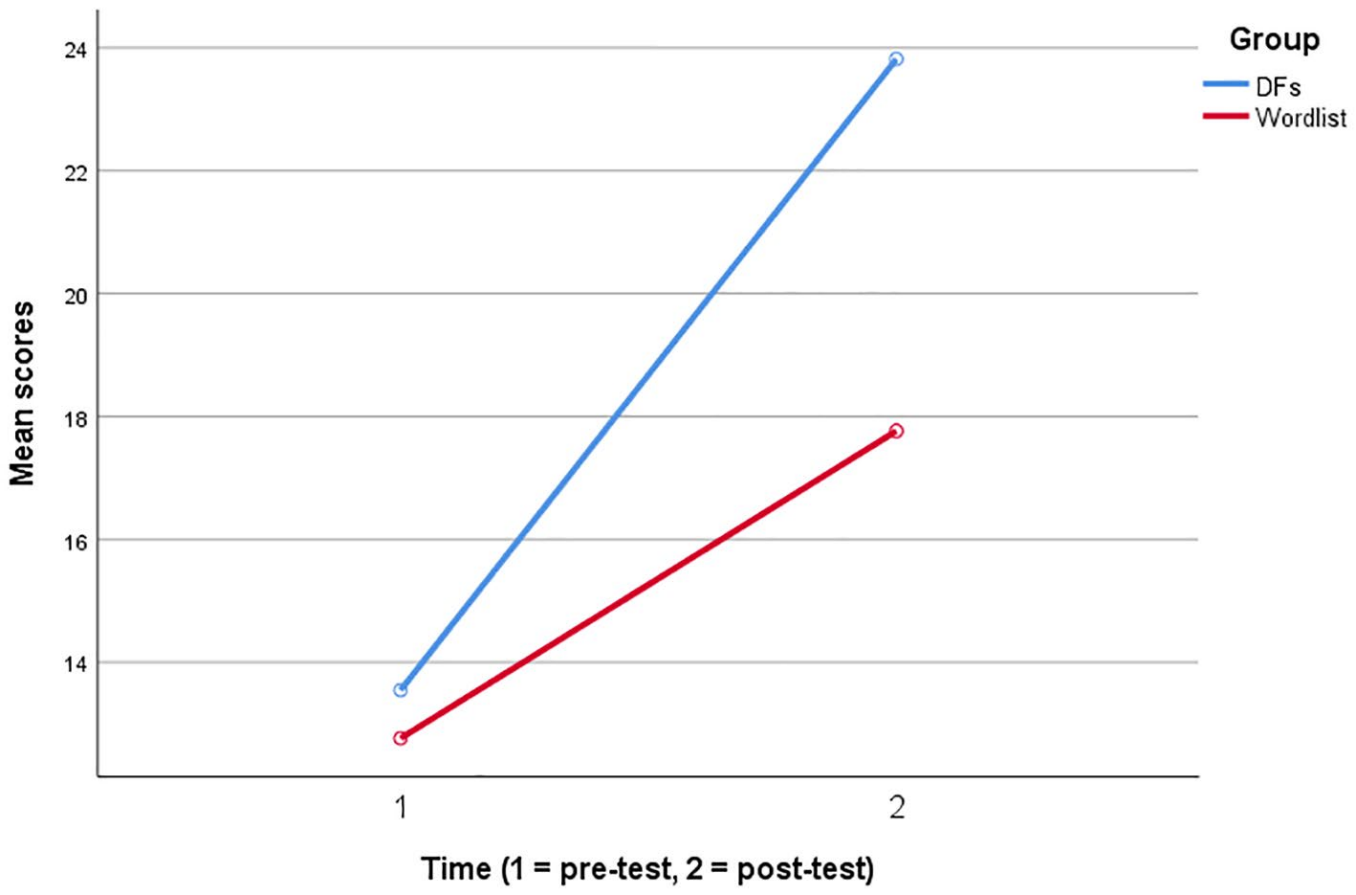
Estimated marginal means for the academic vocabulary knowledge test.

Additionally, Figure 2 visually represents the estimated marginal means for vocabulary test scores. In this regard, the existing differences in the academic vocabulary knowledge of the participants in the experimental (DFs) and control (wordlist) groups were not much noteworthy before the treatments (see Table 2). However, the resulting differences were significant in the post-test, and students who used DFs scored considerably higher than the control learning condition.

## Discussion and conclusion

The first research question examined the contribution of mobile-assisted academic vocabulary learning with digital flashcards as a pedagogical intervention for improving university students’ academic literacy. The findings indicated that although both treatments effectively scaffolded vocabulary learning (Table 2), the participants who used digital flashcards outperformed those in the control group that used traditional materials (i.e., wordlists, Table 3). Consequently, the study provided empirical evidence for the effectiveness of mobile-assisted academic vocabulary for university students. The findings agree with earlier studies that reported similar results (Dizon, 2016; Xodabande et al., 2022; Xodabande and Atai, 2022). The relative effectiveness of mobile-assisted learning might be explained in light of the following considerations. First, digital flashcards provided the participants with a more effective strategy for vocabulary learning. More specifically, the spaced repetition feature enabled them to have multiple encounters with the target words, and such exposure to form-meaning connections facilitated learning academic words (Webb and Nation, 2017; Nation, 2022). Second, learning with digital flashcards involved more retrieval efforts on the part of the participants in the experimental group compared to the control group. As highlighted by previous studies, such mechanisms contribute significantly to improved learning outcomes (Li and Hafner, 2022; Xodabande et al., 2022). Third, it has been observed that integrating digital technologies into language education results in enhanced motivation and learner engagement due to the inherent motivational potential of using technology (Stockwell, 2013; Xodabande and Atai, 2022). Consequently, given the determining role of motivation in language learning success (Dörnyei and Ushioda, 2010), this factor might have contributed to the improved performance of digital flashcards users.

The second research question concerned the impact of digital flashcards on university students’ self-regulatory capacity for academic vocabulary learning. The findings indicated that those participants in the experimental group learned more academic words and developed more self-regulatory capacity for vocabulary learning (Table 3; Figure 1). This finding is in line with the studies that found a similar relationship between mobile-assisted vocabulary learning and improvements in self-regulated learning capacity (Choi et al., 2018; Fathi et al., 2018; Lei et al., 2022). This outcome might have resulted from some factors. First, learning academic words with digital flashcards facilitated the development of some basic self-regulation skills, such as setting realistic goals, managing time and learning resources, and selecting the best strategies for achieving the goals (Zheng et al., 2018; Lei et al., 2022). Second, learning with digital flashcards impacted the participants’ commitment control more effectively than learning from wordlist and accordingly facilitated managing more favorable expectations through learning words in specific sets (Tseng et al., 2006). Third, as highlighted above, digital flashcards bring inherent motivational impacts to vocabulary learning. Considering the role of emotion control in the self-regulatory capacity for vocabulary learning (Ziegler, 2015), it has been argued that increased motivation boosts positive emotionality and minimizes the impacts of negative emotions (MacIntyre et al., 2019). Finally, observed changes in the participants’ self-regulatory capacity might be due to increased metacognitive control over vocabulary learning resulting from digital flashcards (Teng and Zhang, 2022). As mobile-assisted learning provided the participants with affordances to monitor their learning progress and evaluate their performance, they developed more capacity for self-regulated vocabulary learning.

The findings have some implications for teaching academic words and improving EFL university students’ academic literacy. First, as the study indicated, using digital flashcards and mobile-assisted learning effectively result in substantial vocabulary gains. Considering the crucial role of academic vocabulary in scaffolding university students’ literacy development (Coxhead, 2019) and the fact that learning such words is associated with a significant learning burden (Evans and Morrison, 2011), EAP programs might benefit significantly from the integration of mobile-assisted learning into their curricula and instructional processes to address vocabulary learning needs. Second, vocabulary learning *via* digital flashcards seems to be a practical approach to extending learning beyond the classroom. Accordingly, various affordances provided by mobile devices for moving the vocabulary learning activities to anytime and anyplace is specifically noteworthy for EAP teachers (Xodabande and Atai, 2022). Additionally, with the growing importance of self-regulated learning in language education, academic vocabulary learning with digital flashcards might be considered as a pedagogical innovation that facilitates long-term academic literacy development as it builds university students capacity to learn autonomously and to take more responsibility with respect to their learning needs. Finally, as using innovations and technologies for language learning is inherently motivating (Stockwell, 2013), mobile-assisted academic vocabulary learning provides EAP programs with new possibilities to sustain and support university students’ engagement with the learning content. Collectively, these implications highlight the affordances of mobile-assisted vocabulary learning as one of the most recent developments in educational technology. Tailoring this technology to EFL university students’ learning needs through developing their academic vocabulary contributes to the success of EAP programs and helps build their professional identity.

## Data availability statement

The original contributions presented in the study are included in the article/supplementary material, further inquiries can be directed to the corresponding author.

## Ethics statement

Ethical review and approval was not required for the study on human participants in accordance with the local legislation and institutional requirements. The patients/participants provided their written informed consent to participate in this study.

## Author contributions

All authors listed have made a substantial, direct, and intellectual contribution to the work and approved it for publication.

## Conflict of interest

The authors declare that the research was conducted in the absence of any commercial or financial relationships that could be construed as a potential conflict of interest.

## Publisher’s note

All claims expressed in this article are solely those of the authors and do not necessarily represent those of their affiliated organizations, or those of the publisher, the editors and the reviewers. Any product that may be evaluated in this article, or claim that may be made by its manufacturer, is not guaranteed or endorsed by the publisher.

## Footnotes

^1^ https://www.cambridge.es/en/about-us/cambridge-english-corpus

^2^ https://quizlet.com/elearnguy/folders/nawl-50-word-blocks/sets
